# Two cases of 18F‐FDG‐PET/CT positive Schloffer tumor following curative surgery of colon cancer

**DOI:** 10.1002/ccr3.6741

**Published:** 2022-12-21

**Authors:** Hiroki Uehara, Madoka Hamada, Masahiko Hatta, Mitsugu Sekimoto, Yuri Noda, Kotaro Minami, Yumiko Kono, Hiroaki Kurokawa

**Affiliations:** ^1^ Department of Gastrointestinal Surgery Kansai Medical University Hospital Hirakata Japan; ^2^ Department of Surgery Kansai Medical University Hirakata Japan; ^3^ Department of Pathology and Laboratory Medicine Kansai Medical University Hospital Hirakata Japan; ^4^ Department of Radiology Kansai Medical University Hospital Hirakata Japan

**Keywords:** colorectal cancer, Schloffer tumor, tumor extirpation

## Abstract

We report two cases of Schloffer tumor that required resection after radical colon cancer surgery because of suspected lymph node recurrence on contrast‐enhanced (CE) CT and 18F‐FDG‐PET/CT. Case1 is a 69‐year‐old man with sigmoid colon cancer pStage IIA, and case2 is a 61‐year‐old man with descending colon cancer pStage IIIB.

## Graphical abstract

Although the Schloffer tumor is rare, it can be misdiagnosed as a solitary metastatic cancer recurrence. We should be aware of the presence of this tumor and be cautious to avoid futile chemotherapy.

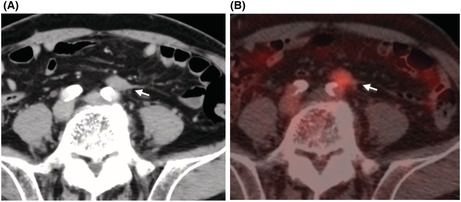

## 1 INTRODUCTION

A rare benign inflammatory tumor occurring after abdominal surgery was first described by the Austrian surgeon Schloffer H. and is recognized as Schloffer tumor.^1^ It is considered a granuloma caused by a foreign body reaction to a substance such as a nonabsorbable thread left in the abdominal cavity at the time of initial surgery. Schloffer tumor after colon cancer surgery has been reported to be challenging to differentiate from the recurrence of the tumor. We have experienced two cases of Schloffer tumors that were suspected of cancer recurrence and required resection because of the growing tumor with ^18^F‐fluorodeoxyglucose Positron Emission Tomography (18F‐FDG‐PET/CT) accumulation. We report our experiences with the Schloffer tumor and review the literature.

## 2 CASE REPORT

### 2.1 Case1

**FIGURE 1:**
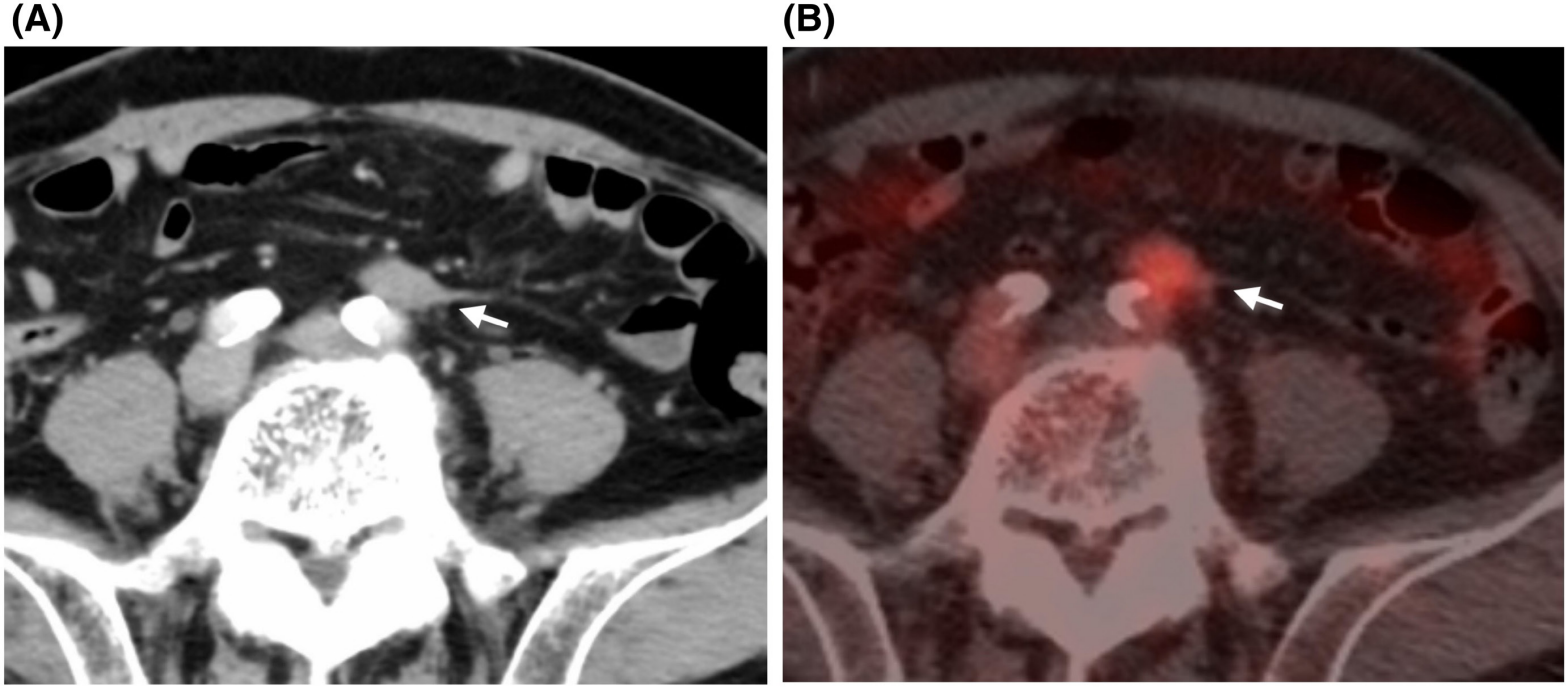
CECT scan and 18F‐FDG‐PET/CT findings in Case1. CECT scan indicates a tumor formation (21 × 12 mm) on the caudal ventral side of the left common iliac artery bifurcation (white arrow, A), corresponding to the SUVmax 5.2 tumor in the 18F‐FDG‐PET/CT scan (white arrow, B).

A 69‐year‐old male had pre‐existing dilated cardiomyopathy, atrial fibrillation, diabetes mellitus, and hyperlipidemia. In April 2021, a patient with sigmoid colon cancer (pT3N0 (0/23) M0 pStage IIA) underwent laparoscopic anterior resection. The postoperative course was uneventful, and he was discharged eighth postoperative day. CECT in April 2022 revealed a tumor (21 × 12 mm) on the caudal ventral side of the left common iliac artery bifurcation, which was not evident in October 2021 (Figure 1A). The SUVmax of the 18F‐FDG‐PET/CT scan at the tumor was 5.2 at the same site (Figure 1B). Blood tests showed no abnormal findings, including CRP, CEA 1.0 (ng/ml), CA19‐9 4.0 (U/ml). He was diagnosed with lymph node recurrence in the anterior aspect of the common iliac artery and underwent surgery in June 2022.

#### 2.1.1 Surgical and pathological findings

**FIGURE 2:**
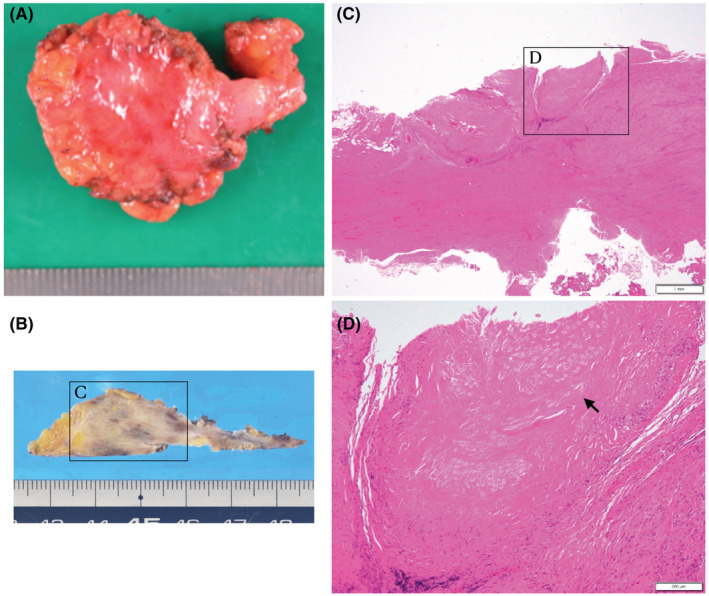
Macroscopic and microscopic findings in Case1. The resected specimen was a hard elastic nodule whose maximum diameter was 35 mm (A, B). Pathological examination reveals scar formation with stromal cell activation (C) surrounding the suture‐like foreign body (D, black arrow). Neither lymph node structures nor findings suggestive of malignancy

The patient underwent tumor extirpation laparoscopically using a 5‐port wound of initial laparoscopic sigmoid colon resection. The tumor was located around the Promontrium, which was thought to be at a common iliac artery bifurcation. We made a peritoneal incision around the tumor from the cephalad to the caudal side of the mass and identified the right branch of a common iliac artery. The tumor was contiguous with the hypogastric nerves, which were removed in combination with the tumor from the medial, mesenteric, and cephalic sides. The resected specimen was a hard elastic nodule with a maximum diameter of 35 mm (Video S1, Figure 2A). Pathological examination revealed scar formation with stromal cell activation surrounding the suture‐like foreign body. Neither lymph node structures nor findings suggest malignancy (Figure 2B–D). The postoperative course was uneventful, and he was discharged on the second postoperative day. He has no evidence of colon cancer recurrence 18 postoperative months after primary surgery.

### 2.2 Case2

**FIGURE 3:**
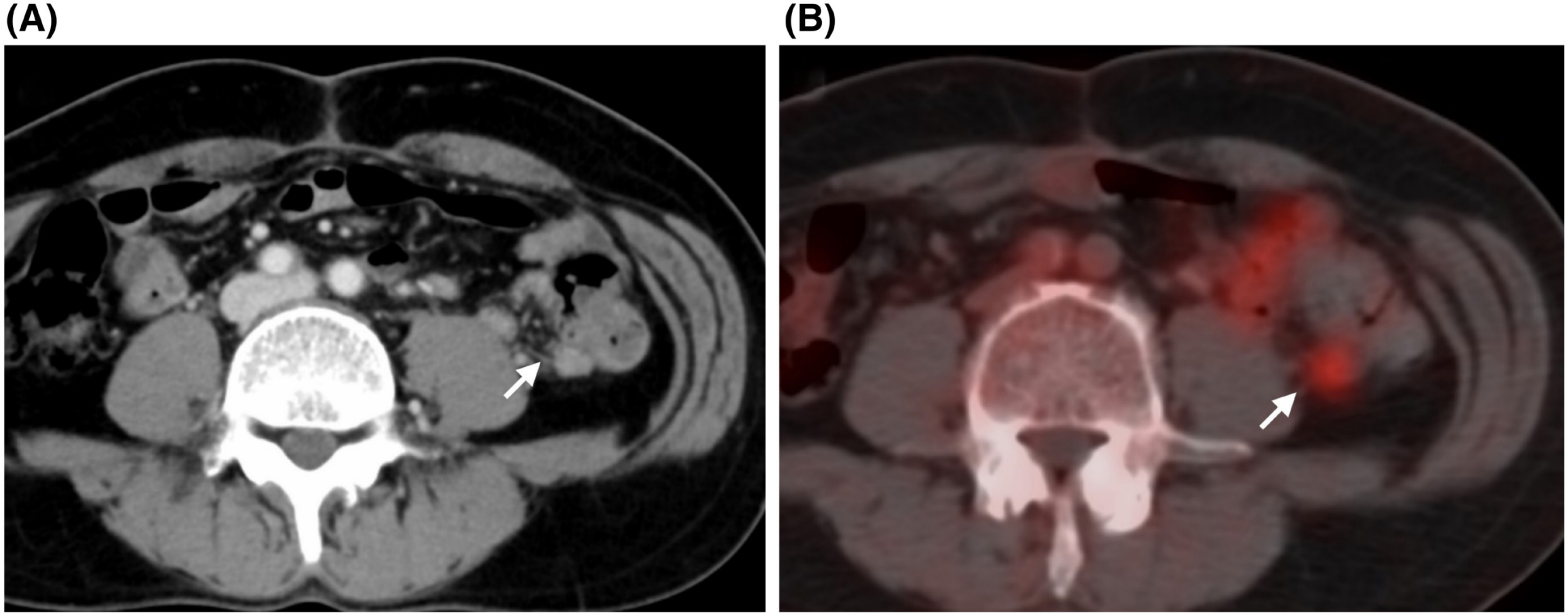
CECT scan and 18F‐FDG‐PET/CT findings in Case2. CECT scan indicates a tumor formation (10 × 11 mm) at the dorsal side of the anastomotic site of descending colon. (A, white arrow), corresponding to the SUVmax 3.4 tumor in the 18F‐FDG‐PET/CT scan (B, white arrow)

A 61‐year‐old male had no significant medical history. He underwent laparoscopic resection of descending colon cancer (pT3N1b (2/14) M0 pStage IIIB) in October 2016. The postoperative course was uneventful, and he was discharged on the eighth postoperative day. Six courses of oral anticancer drugs (UZEL® + UFT®, Taiho Pharmaceutical) were administered as postoperative adjuvant chemotherapy. A CECT scan in July 2017 revealed a tumor (10 × 11 mm) in the mesentery at the dorsal side of the anastomotic site of descending colon (Figure 3A). An August 2017 FGD‐PET scan showed an accumulation of SUVmax 3.4 in the same area (Figure 3B). Blood tests were unremarkable, including inflammatory findings, with CEA 2.1 ng/ml and CA19‐9 < 1.0 U/ml. The patient was diagnosed with recurrent peritoneal dissemination around the anastomosis, and we performed tumor extirpation in September 2017.

#### 2.2.1 Surgical and pathological findings

**FIGURE 4:**
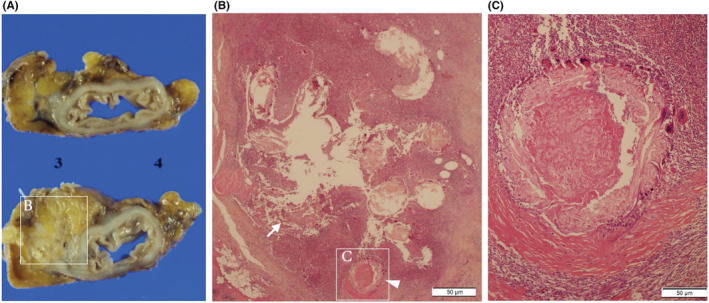
Macroscopic and microscopic findings in Case2. Resected specimen revealed that the tumor was an ill‐defined hard elastic mass, and its size was 50 × 70 × 25 mm (A). Pathological examination reveals an abscess formation (white arrow) was observed (B) around the surgical threads (arrowhead) in the submucosa (C). No neoplastic lesions were identified.

We performed open surgery with a midline incision. The tumor was located dorsally near the previous anastomosis. The descending colon adhered to the parietal peritoneum firmly and was dissected from the lateral side, and the retroperitoneal fascia was also dissected from the inside, preserving the left ureter. We performed tumor resection with descending colon, including the previous anastomotic site (length 6 cm). Resected specimen revealed the tumor was an ill‐defined hard elastic mass, and its size was 50 × 70 × 25 mm (Figure 4A). Pathological examination revealed an abscess formation identical to the Schloffer tumor around the surgical threads in the submucosa. No neoplastic lesions were identified (Figure 4B,C). The postoperative course was uneventful, and he was discharged on the eighth postoperative day.

He has no evidence of colon cancer recurrence 72 postoperative months after primary surgery.

## 3 DISCUSSION

Schloffer tumors usually occur several years after primary surgery, most commonly at the surgical site, but their localization is variable.^2^ Tumors are closely confined to the surrounding tissue and may be well demarcated or develop invasive growths, which may be challenging to differentiate from recurrent lesions of colorectal cancer because of the lack of typical imaging findings.^3^

Twelve cases of Schloffer tumors examined by PET scan after radical surgery of colorectal cancer have been reported in Japanese literature (Table 1). On the contrary, few cases have been reported from western countries.^4^ The duration until tumor detection from the initial surgery is more than 100 months in Shibata, Taki, et al.'s report. Still, most of the reports since 2007 describe they were between 1 and 2 years after surgery. It may be due to the whole‐body enhanced CT scan follow‐up every 6 months after surgery and the health insurance system in Japan allowing patients to receive 18F‐FDG‐PET/CT since 1994. Because the median value of SUVmax was 4.5 (2.7–14.6), it was difficult to distinguish it from cancer recurrence with PET images. Of the 12 cases, 5 cases were suspected of lymph node recurrence, and 6 cases of peritoneal dissemination. Although the silk suture was found in the center of the mass in 5 patients, including our cases, it might not increase the risk of Schloffer tumor because the results of a clinical trial reported by Maehara et al. revealed less SSI, which can be associated with postoperative complications when silk sutures were used than absorbable ones.^5^, ^6^

**TABLE 1:** Reported cases of Schloffer tumor with positive 18F‐FDG‐PET/CT scan after radical resection of colorectal cancer in Japan

No	Year	Author		Primary tumor location	Stage	Duration from the primary surgery (month)	Schloffer tumor location	CEA (ng/ml)	Size (mm)	FDG‐PET	SUVmax	Preoperative diagnosis	Surgery	Suture thread
			Age/Sex											
1	2006	Shibata^10^	73/F	S + Liver metastasis	IV	166	AW	WNL	22	(+)	ND	PD	TX	Suture thread
2	2007	Taki^11^	69/M	R	II	110	Pelvic region	WNL	30	(+)	ND	PD	TX	ND
3	2007	Maeda^12^	47/M	R	IIIa	12	AW	WNL	18	(+)	2.7	PD	TX	Suture thread
4	2013	Kodera^a^	56/F	C + Endometrial cancer	IIIb	2	Pelvic region	WNL	18	(+)	3.6	LN	TX	Silk
5	2013	Kakimoto^13^	57/F	S	IV	19	PALN	7.2	7	(+)	3.82	LN	TX	Suture thread
6	2014	Ohta^14^	62/F	S	IIIa	24	PALN	WNL	25	(+)	ND	LN	Partial resection of the small bowel	Silk
7	2014	Ohta^14^	78/F	A	IIIa	12	Liver (S4)	WNL	25	(+)	ND	Liver metastasis	Partial hepatectomy	Silk
8	2016	Miyake^15^	80/M	A	IIIa	6	Pancreatic head	WNL	20	(+)	5.8	LN	PD	ND
9	2019	Asano^3^	85/M	S + HCC	IIIa	10	AW	WNL	20	(+)	14.6	Port site recurrence	TX	ND
10	2019	Shinjyo^16^	79/M	A	II	20	AW	49.5	25	(+)	5.64	PD	TX in combination with colectomy	Nylon
11	2022	Current1	69/M	S	IIA^b^	12	PALN	WNL	21	(+)	5.2	LN	TX	Silk
12	2022	Current2	61/M	D	IIIB^b^	9	Lymph node in the mesocolon	WNL	10	(+)	3.4	PD	TX in combination with colectomy	Silk

Abbreviations: Primary tumor location: A, ascending colon; C, cecum; D, descending colon; R, Rectum; S, sigmoid colon. Stage: Japanese society for cancer of the colon and rectum, Second English Edition. Schloffer tumor location: AW, abdominal wall. CEA: WNL, within normal limit. FDG‐PET: 18F‐fluorodeoxyglucose positron emission tomography. SUVmax: ND, not described. Preoperative diagnosis: LN, lymph‐node metastasis. Surgery: PD, peritoneal dissemination; PD, pancreaticoduodenectomy; TX, tumor extirpation; Suture thread: ND, not described.

^a^ Literature in Japanese without English abstract.

^b^ TNM Classification of Malignant Tumours, 8th Edition.

According to the Japanese Society for Cancer of the Colon and Rectum Guidelines,^7^ 2019, recurrence after radical surgery of colorectal cancer occurs in 18.7% of patients, with 7.1% having liver metastases, 5.5% lung metastases, 2.0% peritoneal, 2.0% local, 1.1% anastomotic, other 4.8%. Suppose our cases had been recurrent colon cancer lesions. It should have been considered a paraaortic lymph node (PALN) metastasis or peritoneal recurrence, relatively rare as an isolated first recurrent lesion after the radical colorectal cancer surgery. Nozawa et al. reported that eleven patients who underwent surgery for PALN after curative surgery for colon cancer had a significantly better prognosis than those who did not. Still, only six patients of them underwent upfront surgery. The other five cases might be treated with upfront chemotherapy.^8^ Case2 is a case of suspected intra‐mesenteric lymph node recurrence or peritoneal dissemination. Although some reports show the effectiveness of surgery for the complete removal of postoperative peritoneal recurrence,^9^ the significance of the upfront strategy for peritoneal dissemination is unclear. Therefore, upfront chemotherapy may be adopted in some institutions.

In our two cases, only one isolated lesion was detected at the previous surgical site. As Case1 was pN0 and Case2 was pT3N1b, each had a low risk of local recurrence. Although we performed tumor extirpation first as the total biopsies, we considered upfront surgery appropriate for confirming the pathological findings.

We tried to perform en‐bloc resection as a recurrent mass in our surgery. In Case1, the surface of the common iliac artery was exposed from the aorta, and we resected the tumor together with the superior hypogastric nerve. In Case2, the tumor was resected with the descending colon, including the previous anastomosis. Both tumors were hard elastic masses with relatively clear boundaries and the surrounding tissues. As far as we know, there has been no report with an intraoperative video of Schloffer tumor extirpation, and this case may serve as a reference for similar cases.

Schloffer tumors, although rare, should be noted as a tumor hard to be differentiated from a tumor recurrence during the postoperative follow‐up period of colorectal cancer. Considering the stage and nature of the initial surgery, if an isolated lesion is noted on positive PET near the surgical site within two years, we should be aware of the presence of this tumor and be cautious and avoid unnecessary chemotherapy as possible.

## AUTHOR CONTRIBUTIONS

HU and MH are the guarantors of the integrity of the entire study. MH involved in study concepts and design; HU involved in literature research. HU and MH involved in clinical studies. HU, MH, MH, MS, YN, KM, YK, and HK involved in data acquisition. HU and MH involved in manuscript preparation and definition of intellectual content. MH involved in manuscript editing and final version approval.

## FUNDING INFORMATION

No supportive foundations.

## CONFLICT OF INTEREST

The authors have no conflict of interest.

## CONSENT

The patient's written consent form for the published photos was obtained. In addition, written consent for information for research and paper activities was obtained from the patient. This study does not contain identifying information about the patients.

## Supporting information


Video S1

## Data Availability

The data that support the findings of this study are available from the corresponding author, MH, upon reasonable request.
